# CT and MRI multimodality imaging features of endometrial stromal sarcoma: a retrospective single-center analysis of 35 pathologically confirmed cases

**DOI:** 10.3389/fonc.2026.1847673

**Published:** 2026-10-29

**Authors:** Lan Yu, Meizhen Xie, Yan Dong, Xiaoming Zhou, Haiyan Gu, Liling Long

**Affiliations:** 1 Department of Radiology, The First Affiliated Hospital of Guangxi Medical University, Nanning, Guangxi, China; 2 Department of Radiology, The Affiliated Hospital of Qingdao University, Qingdao, China; 3 Department of Radiology, Liuzhou People’s Hospital Affiliated to Guangxi Medical University, Liuzhou, China; 4 Department of Radiology, Affiliated Hospital of Guilin Medical University, Guilin, China; 5 Department of Pathology, The Affiliated Hospital of Qingdao University, Qingdao, China

**Keywords:** CT - computed tomography, endometrial stromal sarcomas, gynecologic oncology, imaging features, diagnosis, MRI

## Abstract

**Objective:**

Endometrial stromal sarcoma (ESS) is an uncommon malignant neoplasm within the female reproductive system. This tumor arises from the stromal cells of the endometrium and ranks as the second most prevalent type of malignant mesenchymal tumor in the uterus, following leiomyosarcoma. It constitutes approximately 1% of all female genital tract cancers and accounts for 3–9% of uterine neoplasms. The clinical specificity of ESS is poor, its imaging manifestations are diverse, and early diagnosis is difficult, relying on pathological confirmation.

**Methods:**

In this retrospective single-center study, we analyzed the CT and MRI imaging findings of 35 pathologically confirmed ESS cases (26 low-grade, 9 high-grade) to identify characteristic imaging features and subgroup differences.

**Results:**

Key findings included the uterine rupture/channel sign (observed in 7 of 21 endometrial-involved cases), honeycomb-like vascular shadows (5 cases), and progressive centripetal homogeneous enhancement (2 cases). Low grade ESS tended to present with larger tumors and more frequent extensive pelvic invasion (mean diameter 10.76 ± 6.58 cm vs. 6.57 ± 1.51 cm, P = 0.007), while high grade ESS more commonly presented with abdominal pain (P = 0.013) and elevated CA19-9 (P = 0.013).

**Conclusion:**

These imaging signs may provide auxiliary clues for preoperative suspicion of ESS; however, definitive diagnosis remains dependent on postoperative pathology and immunohistochemistry. Due to the single-center design, small sample size, and lack of a control group, the diagnostic specificity of these signs requires further validation in multicenter prospective studies.

## Introduction

Endometrial stromal sarcoma (ESS) is a malignant mesenchymal neoplasm arising from endometrial stromal cells. It is the second most common malignant mesenchymal tumor of the uterus, following leiomyosarcoma, accounting for approximately 1% of all female genital tract malignancies and 3%–9% of uterine neoplasms (1–3). ESS lacks specific clinical manifestations, and its imaging features are complex and diverse, resulting in a high preoperative misdiagnosis rate. Definitive diagnosis relies on postoperative pathological examination and immunohistochemistry.

Given the rarity of ESS, existing literature consists mainly of case reports or small case series, and a systematic summary of its multimodality imaging features remains insufficient. Moreover, few studies have performed subgroup comparisons between low-grade and high-grade lesions, which is clinically important given the divergent prognoses and management strategies for these two entities. As noninvasive imaging modalities, CT and MRI can clearly delineate the nature, location, extent, and relationship of the tumor with surrounding tissues, which is valuable for preoperative evaluation and treatment planning. However, the low incidence of ESS limits the statistical power of individual center studies, and methodological rigor becomes paramount.

In this study, we retrospectively analyzed the clinical, pathological, and CT/MRI imaging data of 35 patients with pathologically confirmed ESS at our institution from January 2013 to December 2023. Our objectives were: (1) to summarize the multimodality imaging features of ESS; (2) to perform subgroup comparisons between low-grade and high-grade lesions; and (3) to discuss the diagnostic value and limitations of these imaging findings. We believe this systematic approach will improve radiological recognition of this rare disease and provide evidence-based guidance for preoperative imaging assessment.

## Materials and methods

### Study population

This retrospective single-center observational study was approved by the Medical Ethics Committee of the Affiliated Hospital of Qingdao University, and the requirement for written informed consent was waived.

Inclusion criteria: (1) surgically treated at our hospital between January 2013 and December 2023 with postoperative pathological confirmation of ESS; (2) preoperative pelvic CT or MRI completed within 1 month before surgery; (3) complete clinical, pathological, and imaging data available.

Exclusion criteria: (1) preoperative radiotherapy, chemotherapy, interventional embolization, or hormonal therapy; (2) severe image artifacts compromising interpretation and measurement; (3) concurrent other uterine malignancies; (4) incomplete clinical or pathological data.

### Instruments and methods

Among the 35 cases of ESS, 23 cases underwent CT dynamic enhancement scan, 16 cases underwent MRI examination (including 8 cases with both CT and MRI), and 2 cases only had CT plain scans.

CT examination was performed using GE Lightspeed 16 slice spiral CT machine, GE revolution CT, GE660CT, Siemens Dual Source CT, Siemens Sensing Cardiac64 Spiral CT, Toshiba Aquilion One 640 layer Dynamic Volume CT, and Philips CT 256 row CT machines. Specific parameters: 120kV, 250–350mAs, conventional layer thickness 5mm, scanning range from the top of the diaphragm to the lower edge of the pubic symphysis. CT enhanced scanning uses non-ionic contrast agent (iodine content 300mg/mL), injection flow rate of 3.0mL/s, and total contrast agent amount of 90–100 mL. Scan the arterial phase at 25–30 seconds after injection, scan the venous phase at 60–70 seconds, and scan the delayed phase at 150–180 seconds.

MRI was performed using GE 3.0T MR (Signa HDXT), Siemens 3.0T MR, and Philips 1.5T MR scanners with phased array coils. Patients were supine, with scanning range from the pelvic entrance to the pubic symphysis. Routine MR plain scans included: axial T1WI (TR/TE 600/7.8 ms, slice thickness 6 mm, spacing 1.5 mm, matrix 320 × 192), axial fat-suppression T2WI (TR/TE 3000/85 ms, slice thickness 6 mm, spacing 1.5 mm, matrix 320 × 192), sagittal T2WI (TR/TE 3360/85 ms, slice thickness 6 mm, spacing 1 mm, matrix 320 × 256), coronary fat-suppression T2WI (TR/TE 2460/85 ms, slice thickness 5 mm, spacing 2 mm, matrix 320 × 192), and axial DWI (TR/TE 3575/60.9 ms, slice thickness 6 mm, spacing 1.5 mm, matrix 96 × 130).

Image analysis. Three radiologists with over 5 years of experience in abdominal imaging independently interpreted the images in a blinded manner without knowledge of the pathological results. Prior to image interpretation, all three radiologists underwent a standardized training session on the evaluation criteria and consensus discussion protocol to ensure inter-observer consistency. The evaluation criteria included: (1) tumor location, size, shape, and margins; (2) mass density/signal type (cystic, cystic-solid, or solid); (3) degree of CT enhancement (mild, moderate, or marked relative to the myometrium) and enhancement pattern; (4) MRI signal intensity (low, iso-, or high relative to the adjacent psoas muscle); and (5) metastatic signs, including pelvic effusion, lymph node metastasis, pulmonary metastasis, and hepatic metastasis. Discrepancies were resolved by consensus discussion among the three radiologists. Given the retrospective nature of this study, we acknowledge that formal inter-observer agreement statistics (e.g., Cohen’s kappa) were not performed, which represents a methodological limitation.

Surgical methods and pathological examination. All 35 patients underwent total hysterectomy and bilateral salpingo-oophorectomy. During surgery, the tumor origin, size, number, and abdominopelvic metastatic lesions, as well as lymph node, vascular, and pulmonary involvement, were observed, and peritoneal lavage fluid was collected. Postoperative specimens were routinely processed with paraffin embedding, sectioning, and hematoxylin-eosin (HE) staining. Cytological examination of tumor tissue and peritoneal lavage fluid, as well as immunohistochemical staining, was performed. Disease staging was based on the International Federation of Gynecology and Obstetrics (FIGO) staging criteria for uterine leiomyosarcoma and ESS. According to the 2020 WHO Classification of Female Genital Tumors, ESS was classified into low-grade ESS (LG-ESS) and high-grade ESS (HG-ESS) (4).

Statistical Analysis. Analysis of the data was performed with SPSS 26.0 software for Windows (SPSS Inc., Chicago, IL, USA). Continuous variables were expressed as mean ± standard deviation (SD) and compared between groups using the independent samples t-test. Categorical variables were expressed as frequencies and percentages and compared using the chi-square test or Fisher’s exact test, as appropriate. All statistical tests were two-sided, and the results were considered statistically significant at P < 0.05. The statistical analysis plan was reviewed by a clinical statistician to ensure appropriateness of the methods. Given the relatively small sample size, we acknowledge that the statistical power for subgroup comparisons may be limited, and findings should be interpreted with caution.

## Results

### Patients

Based on the above criteria, 35 patients with ESS were enrolled. The patients ranged in age from 30 to 77 years, with a mean age of 49.2 ± 11.5 years. Clinical manifestations at initial presentation included irregular vaginal bleeding or postmenopausal bleeding in 14 cases, abdominopelvic mass in 10 cases, menorrhagia in 4 cases, abdominal pain in 2 cases, and bilateral pulmonary metastases as the initial symptom in 1 case. The remaining 4 cases were detected during postoperative follow-up for uterine leiomyoma (**Table 1**).

**Table 1:** Clinical manifestations of 35 patients with endometrial stromal sarcoma.

Variable	All ESS (n=35)	Low-grade ESS (n=26)	High-grade ESS (n=9)	P-value
Age (years, mean ± SD)	49.2 ± 11.5	47.8 ± 11.8	53.2 ± 10.0	0.230
Age stratum, n (%)				
<40 years	7 (20.0)	6 (23.1)	1 (11.1)	0.439
40–60 years	21 (60.0)	15 (57.7)	6 (66.7)	0.636
>60 years	7 (20.0)	5 (19.2)	2 (22.2)	0.847
Menopausal status, n (%)				
Premenopausal	28 (80.0)	21 (80.8)	7 (77.8)	0.847
Postmenopausal	7 (20.0)	5 (19.2)	2 (22.2)	0.847
Initial clinical symptom, n (%)				
Irregular vaginal bleeding/postmenopausal bleeding	14 (40.0)	8 (30.8)	6 (66.7)	0.058
Menorrhagia	4 (11.4)	3 (11.5)	1 (11.1)	0.972
Abdominopelvic mass	10 (28.6)*	10 (38.5)*	0 (0.0)*	0.028
Abdominal pain	2 (5.7)*	0 (0.0)*	2 (22.2)*	0.013
Detected on post-leiomyoma follow-up	4 (11.4)	4 (15.4)	0 (0.0)	0.211
Bilateral pulmonary metastasis as initial symptom	1 (2.9)	1 (3.8)	0 (0.0)	0.551

*P < 0.05, statistically significant. Categorical variables were compared using the chi-square test or Fisher's exact test; continuous variables were compared using the independent-samples t-test.

### Laboratory examination

Among the 35 patients, 22 (62.9%) showed mild elevations in serum tumor markers, including elevated CA125 in 14 cases, CA19–9 in 2, CA72–4 in 2, CA15–3 in 1, HE4 in 3, D-dimer in 3, squamous cell carcinoma antigen (SCC-Ag) in 2, AFP in 1, and elevated premenopausal ROMA index in 1. Thirteen patients had all tumor markers within the normal range (**Table 2**).

**Table 2:** Laboratory findings of 35 patients with endometrial stromal sarcoma.

Variable	All ESS (n=35)	Low-grade ESS (n=26)	High-grade ESS (n=9)	P-value
Mild elevation of serum tumor markers, n (%)	22 (62.9)	15 (57.7)	7 (77.8)	0.282
Elevated CA125	14 (40.0)	11 (42.3)	3 (33.3)	0.636
Elevated CA19-9	2 (5.7)*	0 (0.0)*	2 (22.2)*	0.013
Elevated CA72-4	2 (5.7)	1 (3.8)	1 (11.1)	0.418
Elevated CA15-3	1 (2.9)	1 (3.8)	0 (0.0)	0.551
Elevated HE4	3 (8.6)	3 (11.5)	0 (0.0)	0.287
Elevated D-dimer	3 (8.6)	2 (7.7)	1 (11.1)	0.752
Elevated SCC-Ag	2 (5.7)	1 (3.8)	1 (11.1)	0.418
Elevated AFP	1 (2.9)	0 (0.0)	1 (11.1)	0.085
Elevated premenopausal ROMA index	1 (2.9)	1 (3.8)	0 (0.0)	0.551
All markers within normal range	13 (37.1)	11 (42.3)	2 (22.2)	0.282

*P < 0.05, statistically significant. Categorical variables were compared using the chi-square test or Fisher's exact test.

### Pathological findings

Pathology: Among the 35 ESS patients, 26 were low-grade and 9 were high-grade (**Table 3**). Gross pathology showed increased uterine volume in 35 patients, with 16 cases of uterine leiomyoma, 8 cases of uterine adenomyosis, and 6 cases of vascular tumor thrombus (including 2 with intravascular smooth muscle tumor disease and 4 with vascular cancer thrombus).

**Table 3:** Pathological features, grade, and immunohistochemical profiles of 35 patients with endometrial stromal sarcoma.

Variable	All ESS (n=35)	Low-grade ESS (n=26)	High-grade ESS (n=9)	P-value
Pathological grade, n (%)				
Low-grade ESS	26 (74.3)	26 (100)	0 (0.0)	—
High-grade ESS	9 (25.7)	0 (0.0)	9 (100)	—
Concomitant gross lesions, n (%)				
Concomitant uterine leiomyoma	16 (45.7)	12 (46.2)	4 (44.4)	0.929
Concomitant adenomyosis	8 (22.9)	7 (26.9)	1 (11.1)	0.330
Intravascular leiomyomatosis	2 (5.7)	2 (7.7)	0 (0.0)	0.392
Vascular tumor thrombus	4 (11.4)	3 (11.5)	1 (11.1)	0.972
Immunohistochemical positivity, n (%)				
CD10 (+)	35 (100)	26 (100)	9 (100)	—
CD34(+)	5(14.3)	4(15.4)	1(11.1)	1.000
Desmin(+)	3(8.6)	3(11.5)	0(0.0)	0.553
CyclinD1(+)	3(8.6)	2(7.7)	1(11.1)	1.000
Vim(+)	8(22.9)	3(11.5)	5(55.6)	0.015
Caldesmon(+)	6(17.1)	3(11.5)	3(33.3)	0.162
SMA(+)	10(28.6)	8(30.8)	2(22.2)	1.000
P53(+)	8(22.9)	4(15.4)	4(44.4)	0.162
P16(+)	5(14.3)	3(11.5)	2(22.2)	0.586
ER(+)	16(45.7)	14(53.8)	2(22.2)	0.101
PR(+)	13(37.1)	11(42.3)	2(22.2)	0.282
Ki-67 index, median (range)	21(1~90)	16(1~90)	5(5~80)	0.752
FIGO surgical-pathological staging, cases (%)				
Stage IA	7(20.0)	5(19.2)	2(22.2)	0.847
Stage IB	14(40.0)	11(42.3)	3(33.3)	0.636
Stage IIB	3(8.6)	3(11.5)	0(0.0)	0.287
Stage IIIC	2(5.7)	1(3.8)	1(11.1)	0.418
Stage IVA	3 (8.6)	3 (11.5)	0 (0.0)	0.287
Stage IVB	6 (17.1)	3 (11.5)	3 (33.3)	0.135
Advanced stage (FIGO III–IV)	11 (31.4)	7 (26.9)	4 (44.4)	0.329

*P < 0.05, statistically significant. "—" indicates not applicable. Categorical variables were compared using the chi-square test or Fisher's exact test.

Immunohistochemistry: All 35 patients were CD10(+); others included 5 CD34(+), 3 Desmin(+), 3 CyclinD1(+), 8 Vim(+), 6 Caldesmon(+), 10 SAM(+), 8 P53(+), 5 P16(+), 17 ER(+), 15 PR(+). The Ki-67 index ranged from 1% to 90%, with a median of 21% (**Table 3**).

### Postoperative pathological staging

According to FIGO staging criteria, 7 patients were stage IA, 14 stage IB, 3 stage IIB, 2 stage IIIC, 3 stage IVA, and 6 stage IVB. Among low-grade ESS cases, the distribution was 5 IA, 11 IB, 3 IIB, 1 IIIC, 3 IVA, and 3 IVB. Among high-grade ESS cases, the distribution was 2 IA, 1 IB, 0 IIB, 1 IIIC, 0 IVA, and 3 IVB (**Table 3**).

### Imaging features

1. Location and extent. Of the 35 ESS patients, 14 had extensive lesions involving the uterus and adnexa, with normal structure obliteration and abdominal/pelvic metastases. Among the remaining 21 cases, 13 were localized to the uterus with endometrial involvement and myometrial invasion: 3 cases involved only the endometrium, 4 invaded the superficial muscle layer, 3 invaded > 1/2 muscle layer, and 3 invaded the entire uterus. The other 8 cases were confined to the muscle wall (**Table 4**).
2. Tumor size and boundary. In the 14 extensive cases, lesion size was difficult to measure, but maximum diameter exceeded 10 cm. Among the 21 uterine-confined cases, 4 had unclear boundaries, and 17 had clear boundaries with tumor length ranging from 4 to 25 cm (mean 9.7 cm). In 21 endometrial-involved cases, 7 showed outward mass growth destroying the uterine wall (**Figure 1**). Some uterine cavities and bodies exhibited outward rupture, characterized by low density on CT and no enhancement on contrast-enhanced scans (**Figure 2**). MRI showed low signal on T1WI, T2WI, and DWI. Four postoperative ESS patients with uterine fibroids showed tumors in the surgical area, all exhibiting “rupture signs.”
3. Tumor density. Twenty-six lesions were solid, and 9 were cystic-solid. Among these, 3 cystic-solid lesions had extensive necrosis, hemorrhage, and mucinous degeneration; 1 case showed calcification; and 1 had multiple fluid levels with extensive hemorrhage.
4. CT findings. In 23 dynamic CT enhancement scans, 11 cases showed significant arterial phase enhancement in solid areas, with continuous heterogeneous enhancement in venous and delayed phases. Two cases exhibited progressive centripetal enhancement in arterial and portal phases, and uniform enhancement in the equilibrium phase (**Figure 3**).
5. MRI findings. On MRI plain scans, T1WI masses showed mostly equal or mixed signals, with 7 cases having short T1 signals. On T2WI, 2 cases had uniform high signals, and 14 had uneven high or mixed signals. Five cases showed mixed signals with honeycomb-shaped vascular shadows (**Figure 4**). DWI showed high or mixed high signals in 16 cases; combined with T2WI, it helped define tumor boundaries.
6. Metastatic. Abdominal/pelvic fluid accumulation was rare (12 cases, only 1 with tumor cells). Three cases had lung metastases, 1 had right obturator lymph node metastasis, and no liver metastases occurred (**Table 4**).

**Table 4:** Imaging features of 35 patients with endometrial stromal sarcoma.

Variable	All ESS (n=35)	Low-grade ESS (n=26)	High-grade ESS (n=9)	P-value
Tumor origin and extent, n (%)				
Tumor confined to uterus	21 (60.0)	15 (57.7)	6 (66.7)	0.636
Uterus + bilateral adnexa/extensive pelvic invasion	14 (40.0)	11 (42.3)	3 (33.3)	0.636
Endometrial involvement	21 (60.0)	14 (53.8)	7 (77.8)	0.207
Confined to myometrium, no endometrial involvement	14 (40.0)	12 (46.2)	2 (22.2)	0.207
Tumor texture, n (%)				
Solid mass	26 (74.3)	19 (73.1)	7 (77.8)	0.781
Cystic-solid mass	9 (25.7)	7 (26.9)	2 (22.2)	0.781
Characteristic imaging signs, n (%)				
Positive uterine rupture/channel sign	11 (31.4)	7 (26.9)	4 (44.4)	0.329
Honeycomb-like vascular shadows on MRI	5 (14.3)	4 (15.4)	1 (11.1)	0.752
Progressive centripetal homogeneous enhancement	2 (5.7)	2 (7.7)	0 (0.0)	0.392
Metastasis-related findings, n (%)				
Pelvic effusion	12 (34.3)	10 (38.5)	2 (22.2)	0.376
Tumor cells in effusion	1 (2.9)	0 (0.0)	1 (11.1)	0.085
Pulmonary metastasis	2 (5.7)	1 (3.8)	1 (11.1)	0.418
Lymph node metastasis	1 (2.9)	0 (0.0)	1 (11.1)	0.085
Hepatic metastasis	0 (0.0)	0 (0.0)	0 (0.0)	—
Tumor size				
Maximum diameter (cm, mean ± SD)	9.58 ± 5.91	10.76 ± 6.58	6.57 ± 1.51	—
<5 cm	3 (12.0)	3 (11.5)	0 (0.0)	0.287
5–10 cm	14 (56.0)*	7 (26.9)*	7 (77.8)*	0.007
>10 cm	8 (32.0)	8 (30.8)	0 (0.0)	0.058
Extensive pelvic invasion (max diameter >10 cm)	10 (28.6)*	10 (38.5)*	0 (0.0)*	0.028

*P < 0.05, statistically significant. "—" indicates not applicable. Categorical variables were compared using the chi-square test or Fisher's exact test; continuous variables were compared using the independent-samples t-test.

**Figure 1:**
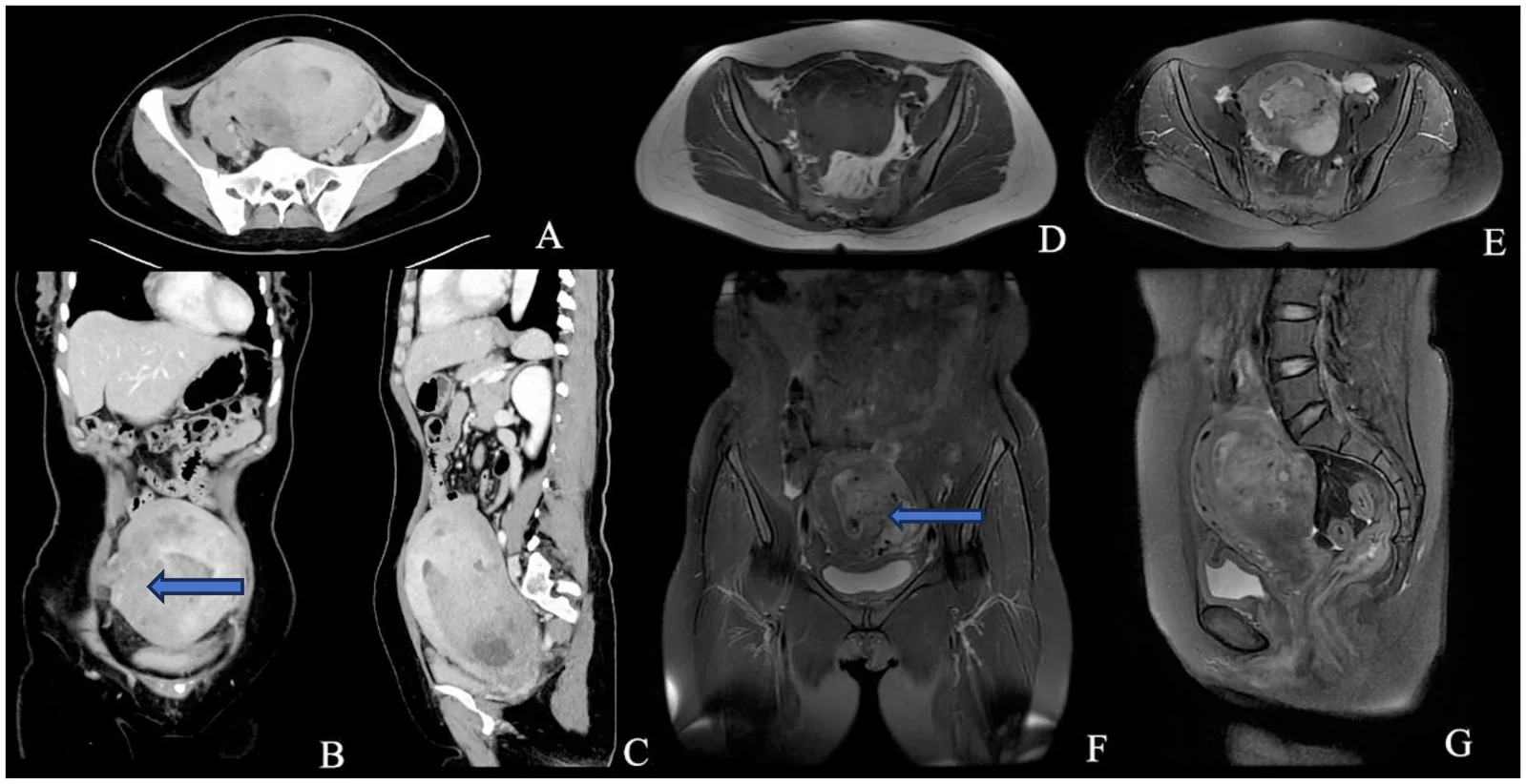
**(A–C)** Patient, 41 years old, with low-grade endometrial stromal sarcoma. CT enhanced scan showed mild uneven enhancement of the uterine mass, with obvious signs of destruction visible on the right posterior wall of the uterine body (arrow). **(D–G)** Patient, 40 years old, with high-grade endometrial stromal sarcoma. MR scans on cross-sectional T1WI, T2WI, sagittal and coronal planes showed left posterior wall of the uterus junction zone, muscle layer erosion, and destruction (arrow).

**Figure 2:**
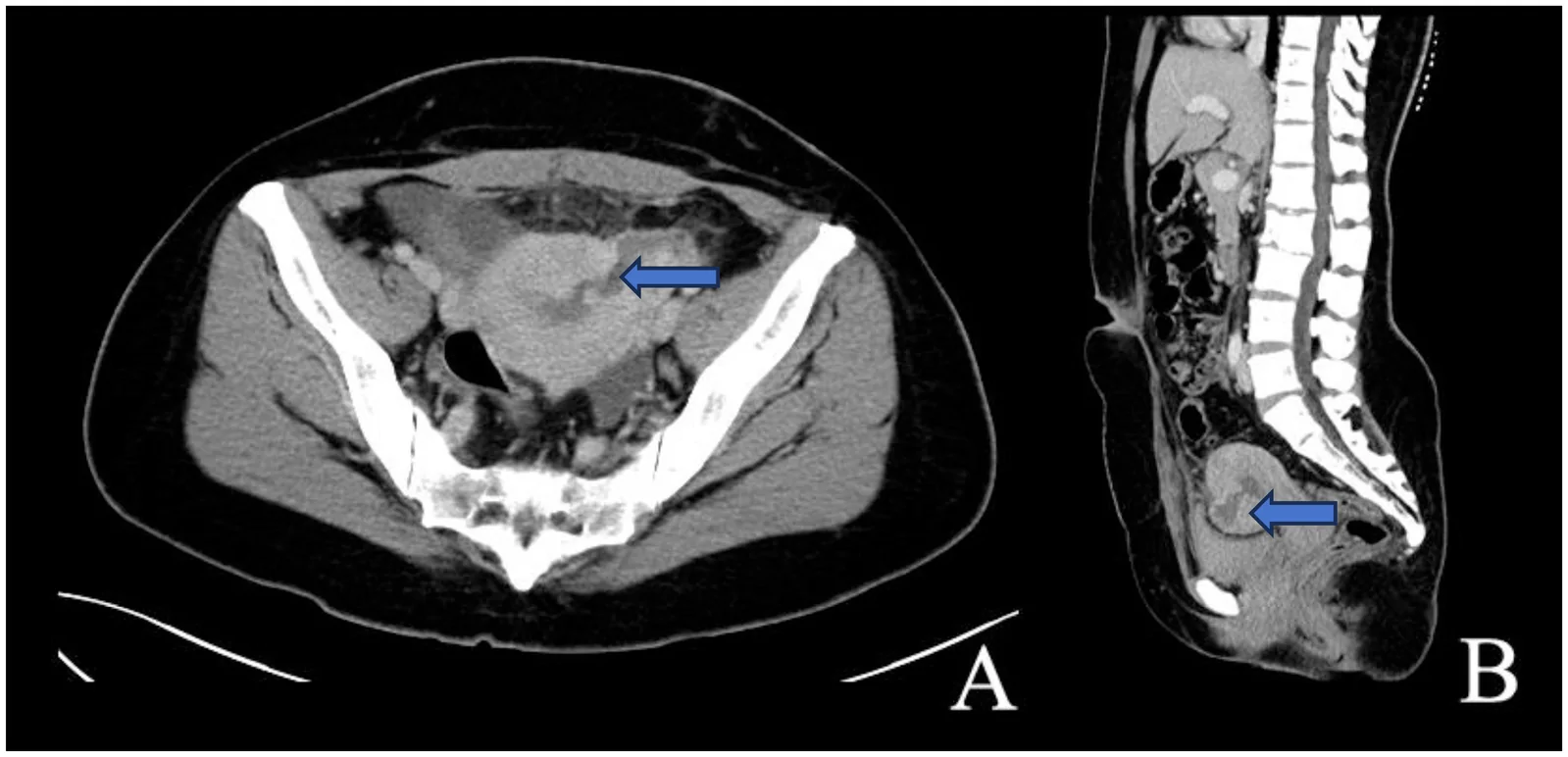
Patients **(A)** and **(B)**, 34 years old, with low-grade endometrial stromal sarcoma. CT enhanced scan shows typical channel and rupture signs (arrows).

**Figure 3:**
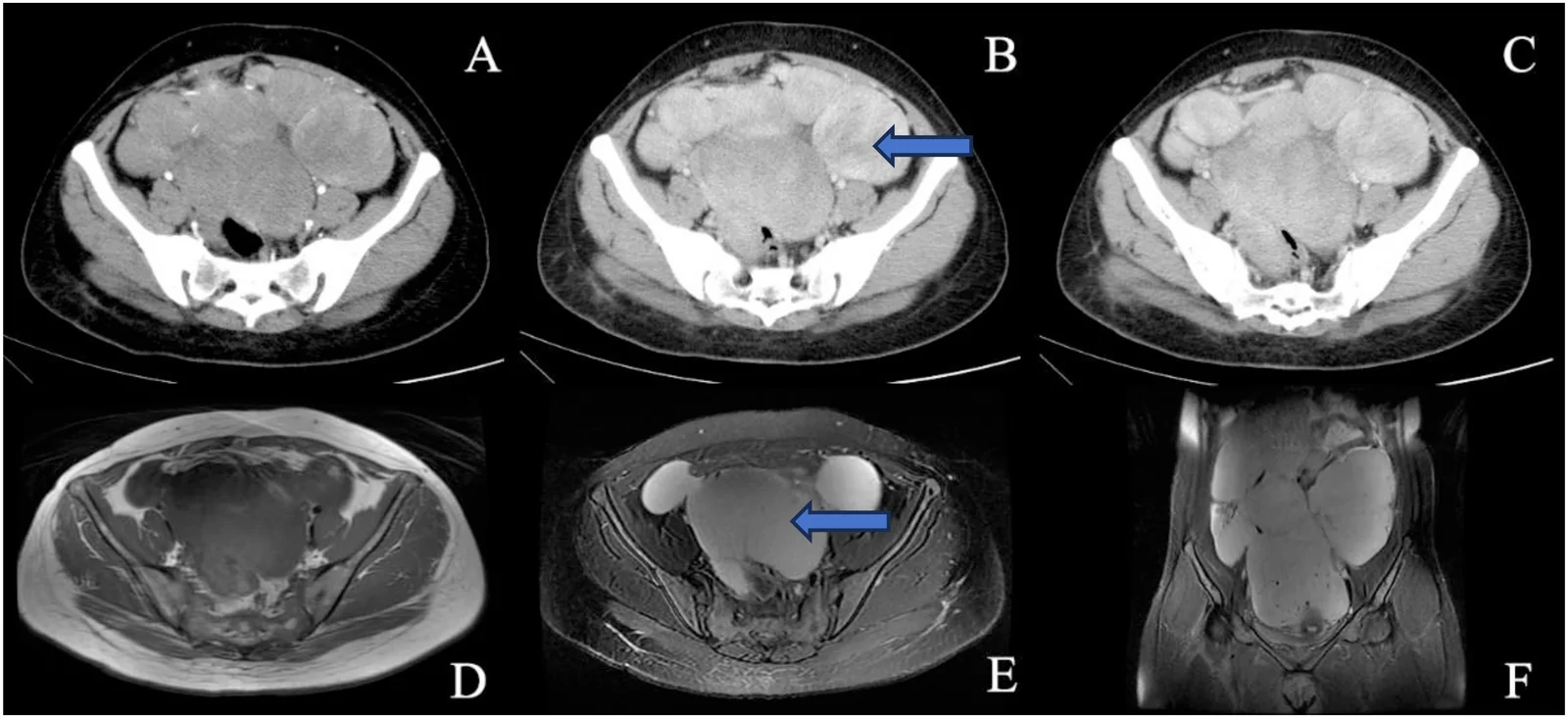
**(A–C)** Patient, 43 years old, with low-grade endometrial stromal sarcoma. CT enhanced scan shows progressive and centripetal enhancement in the arterial and portal phases, and uniform enhancement in the equilibrium phase (arrow). **(D–F)** MR scanning shows uniform T1 high T2 signals (arrow) on cross-sectional T1WI, T2WI, and coronal view.

**Figure 4:**
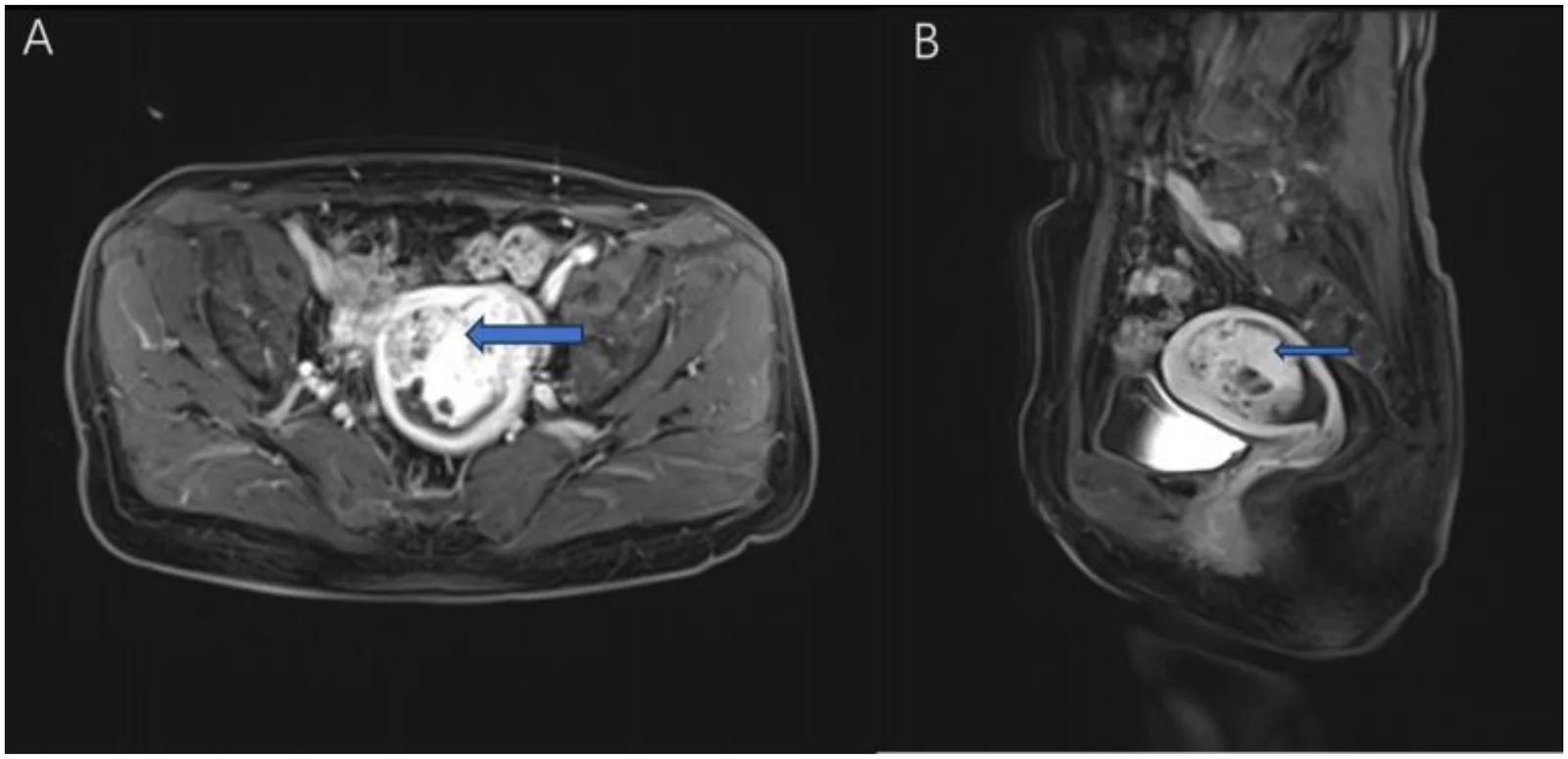
**(A, B)** patient, 7l years old, with high-grade endometrial stromal sarcoma. MRI enhanced scan shows significant heterogeneous enhancement of the uterine cavity mass, limited to the uterine cavity, with honeycomb like tortuous vascular shadows visible (arrow).

### Comparison of clinical, pathological, and imaging features between low-grade and high-grade ESS

A comparison of clinical, serological, pathological, and imaging features between low-grade ESS (n = 26) and high-grade ESS (n = 9) is presented in **Table 1**. High-grade ESS more commonly presented with irregular vaginal bleeding/postmenopausal bleeding as the initial symptom (6/9, 66.7%), whereas low-grade ESS more frequently presented with an abdominopelvic mass (10/26, 38.5%). Statistically significant between-group differences were observed for abdominopelvic mass presentation (P = 0.028) and abdominal pain (P = 0.013). Regarding serological markers, elevated CA19–9 was observed only in the high-grade group (2/9, 22.2%), with a statistically significant difference (P = 0.013).

In terms of imaging features, the mean maximum tumor diameter was 10.76 ± 6.58 cm for low-grade ESS and 6.57 ± 1.51 cm for high-grade ESS. Among tumor size categories, the distribution in the 5–10 cm group differed significantly between grades (P = 0.007). Extensive pelvic invasion (maximum diameter > 10 cm) was observed exclusively in low-grade ESS (10/26, 38.5%), with a statistically significant difference (P = 0.028). No significant between-group differences were found for the uterine rupture/channel sign, honeycomb-like vascular shadows, or progressive centripetal enhancement (all P > 0.05) (**Table 4**).

## Discussion

### Clinical and pathological characteristics

ESS originates from endometrial stromal cells and is a rare mesenchymal malignancy. Its etiology remains unclear but may be associated with endometriosis, tamoxifen exposure, and estrogen levels (5–7). In this study, 8 cases were complicated by adenomyosis and 16 by uterine leiomyoma, consistent with previous reports. ESS tends to form intravascular tumor thrombi; 6 cases in this study had vascular thrombi (including 2 with intravascular leiomyomatosis), reflecting this biological behavior.

ESS has a wide age distribution, with a peak incidence in the fourth to fifth decades (8, 9). The patients in this study ranged from 30 to 77 years of age (mean 49.2 years), including 7 cases under 40 and 6 over 60, which is broadly consistent with the literature. Clinical manifestations are nonspecific, with irregular vaginal bleeding (14 cases) and abdominopelvic mass (10 cases) being the most common, followed by menorrhagia (4 cases), abdominal pain (2 cases), and detection during post-leiomyoma follow-up (4 cases); 1 case presented initially with bilateral pulmonary metastases (10, 11). Laboratory examination most commonly showed mild CA125 elevation (14 cases, 40.0%), which has been rarely reported in previous literature.

According to the 2020 WHO classification, ESS is divided into low-grade and high-grade categories (4). In this study, 26 cases were low-grade and 9 were high-grade. Immunohistochemically, CD10 was diffusely positive in all 35 cases, which is highly specific for ESS (12). ER positivity was seen in 16 cases and PR positivity in 13, indicating hormone receptor expression in a subset of ESS. Subgroup analysis revealed that low-grade ESS more commonly presented with an abdominopelvic mass (P = 0.028), whereas high-grade ESS more frequently presented with abdominal pain (P = 0.013). Elevated CA19–9 was observed exclusively in the high-grade group (P = 0.013). These differences may provide useful clues for preoperative grade assessment.

### Analysis of imaging features

The imaging manifestations of ESS are complex and diverse, with a high preoperative misdiagnosis rate. CT and MRI can clearly demonstrate the nature, location, extent, and relationship of the tumor with surrounding tissues, which may improve preoperative diagnostic accuracy. Through systematic analysis of the imaging data from 35 ESS patients, this study identified the following common suggestive imaging signs:

1. Uterine rupture/channel sign. ESS most commonly arises in the uterine cavity. Owing to its malignant biological behavior, it often demonstrates a tendency to break through the uterine wall and grow exophytically. Imaging reveals incomplete uterine cavity and body architecture, with exophytic tumor growth causing destruction of the uterine wall. Localized outward rupture or channel formation of the uterine cavity or body may be observed. This “rupture/channel sign” appears as low density on CT with no enhancement on contrast-enhanced scans, and as low signal intensity on T1WI, T2WI, and DWI on MRI. Among the 21 cases with endometrial involvement in this study, 7 showed exophytic mass growth with uterine wall destruction, and all 4 ESS patients with prior leiomyoma surgery demonstrated this sign. The presence of this sign, particularly outward rupture or channel formation of the uterine cavity or body, may provide imaging clues suggestive of ESS.
2. Honeycomb-like vascular shadows. In this study, 5 cases with lesions confined to the uterine cavity were initially misdiagnosed as endometrial carcinoma. Careful analysis revealed multiple honeycomb-like vascular shadows in these cases, with some showing marked intraluminal hemorrhage and dilation. This finding may be related to the characteristic pathological arrangement of tumor cells in a spiral pattern around small blood vessels (2). The presence of multiple honeycomb-like vascular shadows in lesions confined to the uterine cavity may help differentiate ESS from endometrial carcinoma (13). However, this sign was seen in only a small number of cases, and some endometrial lesions may also exhibit tiny vascular shadows, so its diagnostic specificity requires further validation.
3. Progressive centripetal homogeneous enhancement. Two cases in this study presented with multiple irregular solid masses in the abdominopelvic cavity, with disappearance of the normal uterine and bilateral adnexal architecture. The solid components showed homogeneous density on CT, iso-signal on T1WI, and homogeneous high signal on T2WI and DWI. Contrast-enhanced CT demonstrated marked enhancement in the arterial phase, followed by continuous centripetal enhancement in the venous and delayed phases, ultimately progressing toward homogeneous enhancement. This enhancement pattern, combined with homogeneous high signal on T2WI and DWI, may also suggest the diagnosis of ESS.
4. Tumor density and signal. ESS masses may present as solid, cystic-solid, or predominantly cystic types (14). In this study, lesions were predominantly solid and cystic-solid, with the vast majority showing marked heterogeneous enhancement and mixed MRI signal. The 14 cases with extensive lesions all had maximum diameters exceeding 5 cm; some were accompanied by hemorrhage, necrosis, or extensive mucinous degeneration, and 1 showed calcification. CT demonstrated masses with heterogeneous density, showing mixed high- and low-density shadows. Septations and mural nodules were visible in non-necrotic cystic areas. Contrast-enhanced CT showed mild heterogeneous enhancement, with “sarcomatoid changes” observed (15, 16). MRI showed mixed high- and low-signal intensity shadows, while T2WI and DWI demonstrated markedly high signal in the solid components, which may help distinguish ESS from uterine leiomyoma (17).

### Differential diagnosis

Due to the nonspecific clinical and imaging features of ESS, preoperative differentiation from the following common uterine lesions is necessary:

1. Uterine leiomyoma. Uterine leiomyoma is the most common benign uterine tumor in women. Typical cases present as well-defined solid masses with iso- or slightly low density on non-contrast CT and homogeneous or heterogeneous enhancement on contrast-enhanced scans, with clear demarcation from the myometrium. On MRI, typical leiomyomas show low signal on T2WI, while degenerated leiomyomas may have heterogeneous signal. Compared with ESS, leiomyomas generally lack uterine wall destruction and the “rupture/channel sign,” rarely show honeycomb-like vascular shadows, and do not form intravascular tumor thrombi. However, large degenerated leiomyomas with necrosis and hemorrhage may overlap with ESS in imaging appearance, making differentiation difficult. This study lacked a concurrent leiomyoma control group, and thus the diagnostic efficacy of the above signs for differential diagnosis could not be quantified.
2. Uterine leiomyosarcoma. Uterine leiomyosarcoma is the most common malignant mesenchymal tumor of the uterus, typically occurring in postmenopausal women. Tumors are often large and prone to necrosis and hemorrhage, with marked heterogeneous enhancement on contrast-enhanced CT and mixed high signal on T2WI. Compared with ESS, leiomyosarcoma is usually positive for SMA and desmin but negative for CD10 on immunohistochemistry, whereas ESS is diffusely positive for CD10. Imaging-wise, leiomyosarcoma less frequently exhibits honeycomb-like vascular shadows and intravascular tumor thrombi, but there is considerable overlap with high-grade ESS. Definitive differentiation relies on pathology and immunohistochemistry.
3. Endometrial carcinoma. Endometrial carcinoma typically occurs in postmenopausal women, presenting with irregular vaginal bleeding. Imaging features include endometrial thickening or an intracavitary mass with enhancement lower than that of the myometrium, high signal on DWI, and reduced ADC values. ESS confined to the uterine cavity needs to be differentiated from endometrial carcinoma: ESS may show honeycomb-like vascular shadows, whereas endometrial carcinoma more commonly manifests as diffuse or focal endometrial thickening and less frequently demonstrates intravascular tumor thrombi or uterine wall rupture signs. Definitive diagnosis requires diagnostic curettage or postoperative pathology.
4. Adenomyosis. Adenomyosis presents with diffuse or focal uterine enlargement, myometrial thickening, widening of the junctional zone on T2WI with multiple small cystic high-signal foci, and enhancement similar to or slightly lower than normal myometrium. Adenomyosis lacks a discrete mass margin, uterine wall destruction, and metastatic signs, making differentiation from ESS relatively straightforward. However, when ESS is complicated by adenomyosis (8 cases in this study), the imaging features of ESS may be masked, increasing diagnostic difficulty.

### Relationship between tumors and surrounding tissues

1. Pelvic effusion. Pelvic effusion is uncommon in ESS (18, 19). In this study, only 12 cases had pelvic effusion, of which 11 were small-volume and only 1 was large-volume with detectable tumor cells. This may be related to the low propensity of ESS for peritoneal metastasis, which helps differentiate it from ovarian malignancies—ovarian cancers are prone to peritoneal metastasis and are often accompanied by substantial abdominopelvic effusion (19).
2. Metastatic pathways. ESS generally metastasizes via the hematogenous route, with lymphatic metastasis being rare (20, 21). In this study, only 1 of 35 patients had pelvic right obturator lymph node metastasis, 2 had bilateral pulmonary metastasis, and no hepatic metastasis was found. Koyama et al. (22) suggested that spread of the mass along blood vessels or ligaments is one of the characteristic features of ESS. Gabal et al. (23) reported a case of ESS invading the heart via the venous system. In this study, 4 cases had venous thrombi, of which 2 were accompanied by intravascular leiomyomatosis.

### Relationship between imaging features and clinicopathological staging

Among the 35 cases in this study, 21 had lesions confined to the uterus (with some endometrial involvement and myometrial invasion), and clinical staging was predominantly stage IA (7 cases) and stage IB (14 cases), including 15 low-grade and 6 high-grade cases. Among the 14 cases with extensive lesions, 3 were stage IIB, 2 stage IIIC, 3 stage IVA, and 6 stage IVB, with 10 low-grade and 4 high-grade cases pathologically. Based on the current imaging findings, clinical staging is related to lesion location and tumor growth duration but has no clear association with pathological type—that is, the more localized the lesion and the shorter the growth duration, the earlier the clinical stage.

### Study limitations

This study has several important limitations that should be acknowledged, as they have implications for the interpretation and generalizability of our findings:

First, this was a single-center retrospective study with a relatively small sample size (35 cases). Single-center studies are inherently subject to selection bias, as patient demographics, referral patterns, and imaging protocols may differ from those at other institutions. The small sample size also limits the statistical power of subgroup analyses, particularly for high-grade ESS (n = 9), where the wide confidence intervals around point estimates indicate considerable uncertainty. These limitations suggest that our findings should be considered hypothesis-generating rather than definitive, and should be validated in larger, multicenter cohorts.

Second, there was no concurrent control group of benign uterine tumors (e.g., leiomyoma, adenomyosis) or other uterine malignancies (e.g., leiomyosarcoma, endometrial carcinoma). Consequently, we were unable to calculate the diagnostic sensitivity, specificity, positive predictive value, and negative predictive value of the imaging signs described in this study, such as the rupture/channel sign and honeycomb-like vascular shadows. The differential diagnostic comparisons presented in the Discussion are primarily based on published literature and our experiential observations rather than quantitative validation from our study cohort. This is a significant limitation, as the diagnostic utility of these signs cannot be rigorously assessed without a control group. Future studies with matched control groups are needed to establish the diagnostic accuracy of these imaging features.

Third, only 16 patients completed comprehensive MRI scanning, which limited the sample size available for MRI-specific analyses. Moreover, due to the retrospective design, the MRI scanning protocols were not uniformly standardized across all 16 patients—scanning parameters varied among the different MRI scanners used (GE 3.0T, Siemens 3.0T, and Philips 1.5T). As a result, we did not perform quantitative ADC analysis or dynamic contrast-enhanced MRI analysis, which could have provided additional diagnostic information. The lack of standardized MRI protocols and quantitative parameters is a notable limitation that may affect the comparability of imaging findings across patients.

Fourth, some patients underwent only non-contrast CT, lacking complete multiphase enhancement data. This precluded a comprehensive assessment of enhancement patterns in all 35 cases and may have introduced information bias. The 23 patients who underwent dynamic CT enhancement scans may not be fully representative of the entire cohort, potentially affecting the generalizability of the CT findings.

Fifth, no predictive model was established, and no external cohort validation was performed. While we identified several imaging features that may be suggestive of ESS, we did not develop a multivariate prediction model that could integrate these features into a clinically useful diagnostic tool. External validation is essential to assess the transportability of any such model to different clinical settings.

Sixth, the retrospective nature of this study means that data completeness and quality were dependent on the quality of documentation in the electronic medical record system. Missing or incomplete data on certain clinical or imaging parameters may have affected the accuracy of our analyses. Additionally, the lack of long-term follow-up data precludes assessment of the relationship between imaging features and patient outcomes, which would be valuable for determining the clinical significance of the identified imaging signs.

In summary, these limitations underscore the need for future multicenter, large-sample, prospective studies with control groups, standardized imaging protocols, and long-term follow-up to validate the imaging features identified in this study and to establish their diagnostic and prognostic value.

## Conclusion

ESS is a rare malignant mesenchymal tumor of the uterus with nonspecific clinical manifestations and diverse imaging features. This retrospective single-center study identified several imaging signs that may provide auxiliary clues for preoperative suspicion of ESS, including the uterine rupture/channel sign, honeycomb-like vascular shadows, and progressive centripetal homogeneous enhancement. Low-grade ESS tended to present with larger tumors and more frequent extensive pelvic invasion, whereas high-grade ESS more commonly presented with vaginal bleeding and abdominal pain. Clinical staging correlates with lesion extent and growth duration but not with pathological grade in this cohort.

However, several important caveats must be emphasized. First, due to the single-center design, small sample size, and lack of a control group, the diagnostic specificity and sensitivity of these imaging signs remain unvalidated. Second, the retrospective nature of the study and the lack of standardized MRI protocols limit the robustness of the MRI findings. Third, without a control group, we cannot determine whether these imaging features reliably distinguish ESS from other uterine tumors such as leiomyoma, leiomyosarcoma, or endometrial carcinoma. Therefore, while these imaging signs may raise suspicion for ESS and prompt further investigation, definitive diagnosis remains entirely dependent on postoperative pathology and immunohistochemistry. Future multicenter prospective studies with control groups and standardized imaging protocols are needed to validate these findings and establish their clinical utility.

## Data Availability

The original contributions presented in the study are included in the article/supplementary material. Further inquiries can be directed to the corresponding author.
